# On Frontal and Temporal Neuralgia

**Published:** 1845-09

**Authors:** 


*On frontal and temporal Neuralgia.*—The external application of croton oil has the great disadvantage of spreading in its effects beyond the place of application, and of causing a disagreeable eruption. This disadvantage can be avoided, and the same effects produced by the application of Ol. cort. hyoscyam. with one grain of acetate of morphia, in the following manner:—A mustard poultice, prepared with cold water, is applied over the situation of the pain, and allowed to remain for an hour; frictions with the above oil are then repeated every two hours. The mustard-poultice is reapplied on the following day, according to circumstances. From four to five applications are generally sufficient to cure the most obstinate neuralgia.—*Ibid, from Dr. Puppi in Asterr. Medic. Wochenschrift.*

